# Frequency of *CDH1* germline variants and contribution of dietary habits in early age onset gastric cancer patients in Brazil

**DOI:** 10.1007/s10120-019-00945-9

**Published:** 2019-03-20

**Authors:** Rodrigo Santa Cruz Guindalini, Marina Cândido Visontai Cormedi, Simone Maistro, Fátima Solange Pasini, Priscila Cristina Abduch Adas Branas, Liliane dos Santos, Gláucia Fernanda de Lima Pereira, Geertruida Hendrika de Bock, Daniela Marques Saccaro, Maria Lucia Hirata Katayama, Sheila Friedrich Faraj, Adriana Safatle-Ribeiro, Ulysses Ribeiro Junior, Maria Del Pilar Estevez Diz, Ana Carolina Ribeiro Chaves de Gouvêa, Roger Chammas, Maria Aparecida Azevedo Koike Folgueira

**Affiliations:** 1 Centro de Investigação Translacional em Oncologia (CTO), Instituto do Cancer do Estado de Sao Paulo (ICESP), Hospital das Clinicas da Faculdade de Medicina da Universidade de Sao Paulo (HCFMUSP), Av Dr Arnaldo, 251, 8th floor, Cerqueira César, São Paulo, SP 01246-000 Brazil; 2 CLION, Grupo CAM, Salvador, BA Brazil; 3 Department of Epidemiology, University of Groningen, University Medical Center Groningen, Groningen, The Netherlands; 4 Instituto do Cancer do Estado de Sao Paulo (ICESP), Hospital das Clinicas da Faculdade de Medicina da Universidade de Sao Paulo (HCFMUSP), São Paulo, SP Brazil

**Keywords:** *CDH1*, Diet, Hereditary diffuse gastric cancer, E-cadherin, Risk factors

## Abstract

**Introduction:**

The contribution of *CDH1* germline variants to gastric cancer burden among young adults is unknown in Brazil. We aimed to evaluate the frequency of *CDH1* germline variants and the diet/lifestyle habits in early age onset gastric cancer (EOGC, ≤ 55 years old) patients.

**Methodology:**

From 2013 to 2015, a total of 88 unrelated and consecutive patients diagnosed with EOGC were enrolled. All *CDH1* exons and intronic boundaries were sequenced, and large genomic rearrangements were screened by MLPA. *CDH1* transcription analysis was performed for variants that could potentially induce an effect on splicing. The diet and lifestyle habits of EOGC patients were compared to Brazilian population diet and lifestyle, obtained from governmental databases.

**Results:**

Of 88 patients, the mean age at EOGC diagnosis was 39 years and 55% fulfilled the criteria for hereditary diffuse gastric cancer. The majority of the tumors were diffuse (74%) and poorly differentiated (80%). In total, 4 novel missense variants of uncertain significance (VUS) were identified: c.313T>A, c.387G>T, c.1676G>A, and c.1806C>A. The MLPA results revealed no rearrangements and *CDH1* transcription analysis for variants of interest were inconclusive. EOGC patients had a higher red (OR:2.6, 95%CI:1.4–4.9) and processed (OR:3.1, 95%CI:1.6–6.0) meat intake and higher fruit consumption (OR:0.4, 95%IC:0.3–0.7) compared to eating habits of the Brazilian population.

**Conclusions:**

No unequivocal pathogenic germline *CDH1* variants were identified in Brazilian EOGC patients. Dietary habits may be associated with the EOGC development.

**Electronic supplementary material:**

The online version of this article (10.1007/s10120-019-00945-9) contains supplementary material, which is available to authorized users.

## Introduction

Gastric cancer is diagnosed in approximately 1 million people globally, is responsible for 780.000 deaths each year, and is currently the third leading cause of cancer death worldwide [1]. Its incidence shows remarkable geographical variation. More than 70% of the cases occur in the developing countries, with the highest incidence rates observed in East Asia, Latin America, Central and Eastern Europe; and the lowest incidence in Africa and Northern America [2]. Brazil is considered a middle-/high-incidence country, with 21.290 new cases expected in 2018 [3].

Although environmental and lifestyle factors—such as *Helicobacter pylori* infection, obesity, tobacco, alcoholic drinks, and foods preserved by salting and processed meat—are major contributors to the etiology of this disease, familial aggregation is observed in approximately 10% of gastric cancer cases which are thought to be hereditary. Overall, only 1–3% arise as a result of inherited cancer predisposition syndromes [4]. Among the hereditary forms, the most important genetic mechanism is associated with germline mutations in the *CDH1* gene (E-cadherin gene type 1, epithelial cadherin, and OMIM #192,090), which encodes the protein called E-cadherin that is a transmembrane calcium-dependent cell-adhesion molecule involved in cell-junction formation and the maintenance of epithelial integrity [5]. *CDH1* germline pathogenic mutations cause hereditary diffuse gastric cancer syndrome (HDGC) [6, 7].

Even though gastric cancer remains a major public health issue in South America, where countries in the region have some of the highest mortality rates worldwide, there is a lack of research focusing on risk factors influencing gastric cancer burden, specially those involving genetic inheritance. To date, only five germline variants of *CDH1* were described in gastric cancer patients in South America: 4 in Brazil [8–10] (c.185G>T, c.1018A>G, c.1763_1764delTG, c.1023T>G), 1 in Argentina [11] (c.1913G>A), and 1 in Colombia [6] (c.2245C>T). In general, germline *CDH1* mutations were identified in subjects with a strong family history of diffuse gastric cancer or lobular breast cancer. However, a combined analysis of 264 sporadic early age onset gastric cancer (EOGC) cases from low-incidence countries found that 2.3% of the subjects had a *CDH1* germline pathogenic mutation [12], highlighting the importance to investigate hereditary cancer in this subpopulation.

Given the fact that inherited risk factors involved in the development of gastric cancer in Brazil are largely unexplored, we investigated the incidence and mutational spectrum of germline *CDH1* variants as well as environmental and lifestyle risk factors in Brazilian early onset gastric cancer patients.

## Materials and methods

### Study population

Consecutive and unrelated patients diagnosed up to 55 years old with gastric carcinoma, except those with neuroendocrine carcinoma, were invited to participate in the IRB approved study at Instituto do Câncer do Estado de São Paulo-Hospital das Clínicas da Faculdade de Medicina da Universidade de São Paulo (ICESP-HCFMUSP), Brazil. All patients who agreed to participate signed an informed consent, underwent genetic counseling, and donated blood for *CDH1* complete sequencing. Personal and familial histories of cancer from EOCG patients were collected through a structured questionnaire. In addition, medical records were reviewed for all participants. Using the personal and family history data, probands were categorized based on whether they met International Gastric Cancer Linkage Consortium (IGCLC) criteria [13–16].

### DNA extraction from blood

DNA was extracted from 8 mL of whole blood using the Biopur Kit Mini Spin Plus (Mobius Life Science, Pinhais, PR, Brazil) and Illustra Blood GenomicPrep Mini Spin Kit (GE Healthcare Bio-Sciences, Pittsburgh, PA, USA/28-9042-64), following the instructions of the manufacturer.

### Polymerase chain reaction (PCR) amplification, Sanger sequencing, and multiplex ligation-dependent probe amplification of *CDH1* gene

Briefly, all exons and intron boundaries of *CDH1* gene were amplified and sequenced in both forward and reverse directions. Primers and conditions are described in Supplementary Table 1. Sequences obtained were visualized by Chromas (v2.33; Technelysium Pty, Ltd., Eden Prairie, MN, USA) and by Mutation Surveyor software (v3.20, SoftGenetics LLC, State College, PA, USA). All patients’ samples were submitted to Multiplex Ligation-Dependent Probe Amplification - MLPA technique (SALSA^®^ MLPA^®^ P083-050R probemix; MRC-Holland, Amsterdam, The Netherlands), to investigate the presence of large rearrangements, as described in the Supplementary Materials.

### *CDH1* sequencing analysis and reporting criteria

All variants were named according to *CDH1* sequence available at GenBank (NM_004360.4) using the nomenclature reported by the Human Genome Variation Society, HGVS (http://www.hgvs.org). The variants were searched for their classification in two publicly accessible databases: Leiden Open Variation Database (LOVD v3.0 build 13) and CLINVAR [17], freeze January 2018.

### Allele frequencies of *CDH1* variants

The difference of the prevalence of identified germline variants was evaluated in publicly available population datasets (ExAC—10.1038/nature19057) and in 609 Brazilian controls [18].

### In silico analysis

Missense variants were analyzed in the following in silico prediction models: Polymorphism Phenotyping (PolyPhen; v2.2.2) [19], Sorting Intolerant From Tolerant (SIFT; v1.0.3) [20], Align-GV/GD [21], MutationTaster2 [22], and Protein Variation Effect Analyzer (Provean; v1.1) [23]. To check for intronic and exonic variants leading to potential splicing defects, the following prediction tools were used: Human Splicing Finder [24], Neural Network (NNS, v0.9) [25], MaxEntScan (MES) [26], and NetGene2 (NG2, v2.42) [27].

### Variant classification

The variants were classified according to recommendations of the American College of Medical Genetics and Genomics in: pathogenic, likely pathogenic, benign, likely benign, and variant of uncertain significance (VUS) [28].

### RNA extraction from paraffin-embedded samples and characterization of the impact on splicing for *CDH1* variants

Briefly, samples harboring *CDH1* variants of interest were chosen for RNA extraction, further cloning, and sequencing, as shown in the Supplementary material.

### Diet and lifestyle habits analysis

Diet and lifestyle information from EOCG patients was collected through a structured questionnaire. The exposure to smoking and alcohol intake was assessed through categories (never, former, and present use). The food intake of fruits, vegetables, leaves, red meat, processed meat, and salty food was assessed by intake frequency categories (less than once a week, once to twice a week, three-to-five times a week, and six-to-seven times a week). To estimate the association of these factors and gastric cancer, information from Brazilian population diet and lifestyle databases was used as a control group. This information was retrieved from the following online public databases: Instituto Brasileiro de Geografia e Estatística (IBGE) [29], Sistema de Vigilância Alimentar e Nutricional (SISVAN) [30], and the Instituto Nacional de Ciência e Tecnologia para Políticas Públicas do Álcool e outras drogas (INPAD) [31]. These governmental data were obtained through population surveys from 2008 to 2015, and is representative of the Brazilian population. These surveys provided information regarding smoking habits, alcohol consumption, and food-intake frequency through similar categories as used in our patients´ questionnaires. The associations between exposures to diet and lifestyle factors and gastric cancer were estimated using logistic regression and calculating the odds ratios (ORs) and 95% confidence intervals (CIs). For this, SPSS version 20 was used. *P* < 0.05 was considered as statistically significant.

## Results

### Population characteristics

From October 2013 to August 2015, 93 consecutive and unrelated patients diagnosed with gastric cancer ≤ 55 years were enrolled. However, two patients were not successful in collecting blood and three patients were excluded, because the diagnosis changed after pathology review by a gastrointestinal pathologist at ICESP-HCFMUSP (two patients were diagnosed with neuroendocrine tumors, and in one patient, the malignancy was not confirmed in the histological study review).

The characteristics of the remaining 88 EOGC patients are shown in Table 1. The mean age at diagnosis was 39 years. Patients were born in all regions of Brazil; most of them were originally from Southeast (50%) and Northeast (38.6%) regions (Supplementary Fig. 1). There was no difference between sexes. The majority of the tumors were diffuse (74%), poorly differentiated (80%), and located in the middle and distal-third of the stomach (67%). Most patients were diagnosed with locally advanced disease (27.3%) or metastatic (39.8%) disease. More than half underwent gastrectomy (58%) and about 28% initially treated with curative intent, had tumor recurrence. The *H. pylori* infection was confirmed in 6 out of 32 cases (infection status was unknown in 56 cases).

**Table 1:** Clinical–pathological characteristics of patients (*n* = 88)

Age at onset (years, range)	39 (20–55)
*Sex*	
Female	45 (51,1%)
Male	43 (48,9%)
*Region of birth (regions of Brazil)*	
North	4.6%
Northeast	38.6%
Central West	2.3%
Southeast	50.0%
South	3.4%
Foreigner	1.1%
*Clinical stage at diagnosis* (%)	
I	14 (15.9%)
II	14 (15.9%)
III	24 (27.3%)
IV	35 (39.8%)
Unknown	1 (1.1%)
*Tumor location*	
Cardia	17 (19%)
Non-cardia	59 (67%)
Unknown	12 (14%)
*Lauren classification*	
Diffuse	65 (74%)
Intestinal	4 (4%)
Mixed	6 (7%)
Others/unknown	13 (15%)
*Tumor grade*	
Poorly differentiated	70 (80%)
Moderately differentiated	4 (4%)
Well differentiated	1 (1%)
Unknown	13 (15%)
*H. pylori infection*	
Yes	6 (7%)
No	26 (29%)
Unknown	56 (64%)
*Gastrectomy*	
Yes	51 (58%)
No	37 (42%)
*Tumor recurrence*	
Yes	15/53 (28%)
No	38/53 (72%)
*Cancer family history*	
1st or 2nd degree relatives with gastric cancer < 50 years	7 (8%)
1st or 2nd degree relatives with gastric cancer > 50 years	13 (15%)
1st or 2nd degree relatives with breast cancer < 50 years	6 (7%)
1st or 2nd degree relatives with breast cancer > 50 years	1 (1%)
IGCLC criteria (1999) [13]	0 (0%)
IGCLC criteria (2010) [15]	48 (55%)
IGCLC criteria (2015) [16]	48 (55%)
Familial Gastric Cancer criteria [14]	59 (67%)

The cancer family history of the study participants is described in Table 1. In total, 23% and 8% of the probands had 1st or 2nd degree relatives diagnosed with gastric and/or breast cancer, respectively. Nevertheless, the probands were not able to confirm neither inform the histologic subtype (e.g., diffuse or lobular) of those cancers diagnosed in their relatives. Because of that, none met the classical criteria postulated in 1999; however, the 2010 and 2015 IGCLC criteria were fulfilled by 55% of the patients; 47% met the criteria due to the diagnosis of diffuse gastric cancer before 40 years of age.

### Complete *CDH1* sequencing

Among these patients, 24 distinctive germline variants were identified (Table 2), including 5 (20.8%) missense, 6 (25%) synonymous, and 13 (54.2%) intronic variants. No large rearrangements were detected through MLPA.

**Table 2:** *CDH1* germline variants

Location	Genomic position	Codon	HGVS c.	HGVS p.	Type	*n*	dbSNP	AF probands	AF ExAC	AF Bras	Classification
Intron 1	Chr16:68.771.372		c.48+6C>T		I	78	rs3743674	0.443	0.811	NA	B–P
Intron 1	Chr16:68.772.141^a^		c.49-59G>T		I	9	NA	0.051	0.000	NA	B
Intron 2	Chr16:68.772.369^a^		c.163+57G>A		I	16	NA	0.0909	NA	NA	B
Intron 2	Chr16:68.772.371^a^		c.163+59G>C		I	5	NA	0.02840	NA	NA	B
Exon 3	Chr16:68.835.722^a^	105	c.313T>A	S105T	M	1	NA	0.0057	0.000	NA	VUS
Exon 3	Chr16:68.835.733	108	c.324A>G	R108R	S	1	rs116542018	0.0057	0.001038	0.0049	LB
Exon 3	Chr16:68.835.754	115	c.345G>A	T115T	S	1	rs1801023	0.0057	0.004	0.005	B
Exon 3	Chr16:68.835.796^a^	129	c.387G>T	Q129H	M	1	NA	0.0057	0.00001	NA	VUS
Intron 3	Chr16:68.835.823		c.387+27C>T		I	1	rs33932809	0.0057	0.001159	0.0041	LB
Intron 3	Chr16:68.842.283		c.388-44G>A		I	1	rs368884824	0.0057	0.000396	NA	LB
Intron 4	Chr16:68.842.480		c.531+10G>C		I	4	rs33963999	0.023	0.038	0.029	B–P
Intron 4	Chr16:68.842.578		c.532-18C>T		I	1	rs200673941	0.0057	0.003	0.003	LB
Intron 6	Chr16:68.845.571		c.833-16C>G		I	2	rs33984587	0.011	0.001	0.004	LB
Exon 11	Chr16:68.853.293^a^	559	c.1676G>A	S559N	M	1	NA	0.0057	0.000	NA	VUS
Exon 12	Chr16:68.855.998^a^	602	c.1806C>A	F602L	M	1	NA	0.0057	0.000	NA	VUS
Exon 12	Chr16:68.856.041	617	c.1849G>A	A617T	M	4	rs33935154	0.023	0.004	0.007	LB
Exon 12	Chr16:68.856.088	632	c.1896C>T	H632H	S	7	rs33969373	0.040	0.011	0.039	B–P
Intron 12	Chr16:68.857.289		c.1937-13T>C		I	13	rs2276330	0.074	0.105	0.075	B–P
Exon 13	Chr16:68.857.441	692	c.2076T>C	A692A	S	78	rs1801552	0.443	0.655	0.344	B–P
Intron 13	Chr16:68.857.544^a^		c.2164+16insA		I	6	rs34939176	0.034	0.045	NA	B–P
Exon 14	Chr16:68.862.165	751	c.2253C>T	N751N	S	8	rs33964119	0.045	0.040	0.038	B–P
Intron 15	Chr16:68.863.710		c.2439+10C>T		I	1	rs35236080	0.0057	0.001	NA	LB
Intron 15	Chr16:68.863.756^a^		c.2439+56T>G		I	3	NA	0.017	0.000	NA	B
Exon 16	Chr16:68.867.387	878	c.2634C>T	G878G	S	4	rs2229044	0.023	0.009631	0.03	B–P

*AF* allele frequency; AF Bras; allele frequency reported in 609 Brazilian controls published by Brito et al. [18]; *B* benign, *B–P* benign–polymorphism, *Chr* chromosomes, *Classif* classification, *dbSNP* single-nucleotide polymorphism database, *EXAC* exome aggregation consortium, *HGVS* human genome variation society, *I* intronic variant, *LB* likely benign, *M* missense, *n* number, *NA* not available, *S* synonymous, *VUS* variant of uncertain significance

^a^variant not described previously

Of the 24 variants, 9 were novel (c.49-61T>G, c.163+57G>A, c.163+59G>C, c.313T>A, c.387G>T, c.1676G>A, c.1806C>A, c.2164+16insA, and c.2439+56T>G) and 8 were classified as polymorphisms, because the population frequencies were greater than 1% in the ExAC databank (c.48+6C>T, c.531+10G>C, c.1896C>T, c.1937-13T>C, c.2076T>C, c.2164+16insA, c.2253C>T, and c.2634C>T).

Excluding the eight variants classified as polymorphisms, 32 patients presented *CDH1* germline variants (regardless of their pathogenicity), corresponding to 36.4% of the cases (Supplementary Table 2).

### In silico analyses of the missense variants

In total, five missense variants were identified; four of them were never previously reported: c.313T>A, c.387G>T, c.1676G>A, and c.1806C>A. In silico analyses of missense substitutions using five different prediction tools have shown conflicting results. All variants were considered benign by SIFT, PROVEAN, and PolyPhen-2 algorithms. On the other hand, Mutation Taster considered all the variants as potential disease causing. No variant has reached the maximum score of pathogenicity by the Align-GV/GD software, but the c.313T>A, c.387G>T e c.1676G>A achieved high scores (Table 3).

**Table 3:** In silico analysis, previously described functional analysis and databases entries of *CDH1* germline missense variants

Variants	PolyPhen-2	SIFT	Mutation taster	PROVEAN	Align-GV/GD	Functional analysis	CLINVAR	LOVD
	(0–1)	(0–1)	(prob)	(-13–4)	(C0-C65)			
c.313T>A	Benign	Tolerated	Disease causing	Neutral 250	C55	NA	NA	NA
(S105T)	(0.035)	(0.41)	(− 58)	(− 0.80)				
c.387G>T	Benign	Tolerated	Disease causing	Neutral 250	C15	NA	NA	NA
(Q129H)	(0.000)	(0.36)	(− 24)	(0.05)				
c.1676G>A	Benign	Tolerated	Disease causing	Neutral 250	C45	NA	NA	NA
(S559N)	(0.000)	(− 1)	(− 46)	(1.94)				
c.1806C>A	Benign	Tolerated	Disease causing	Neutral 250	C15	NA	NA	NA
(F602L)	(0.140)	(0.2)	(− 22)	(0.07)				
c.1849G>A	Benign	Tolerated	Disease causing	Neutral 250	C55	Mild consequence [34]	Conflicting interpretation	+/
(A617T)	(0.040)	(0.19)	(− 58)	(− 0.72)				

*LOVD* Leiden open variation database, *NA* not available, *PolyPhen-2* v2 polymorphism phenotyping, *PROVEAN* protein variation effect analyzer, *SIFT* sorting intolerant from tolerant

+/, Responsible for depositing the variant in LOVD indicates that it affects function, but the curator of LOVD did not classify this variant as pathogenic

The missense mutation c.1849G>A has been previously reported. It was identified in four patients in our study: four women with diffuse gastric cancer diagnosed at 31, 35, 43, and 48 years. This variant was first described as a pathogenic somatic mutation in an endometrial [32] tumor and as a pathogenic germline mutation in a diffuse gastric cancer patient [33]. This variant is localized in the extracellular portion of E-cadherin, affecting a conserved sequence encoding one of the calcium-binding motifs. These calcium-binding motifs are functionally important, because the presence of calcium ions stabilizes the active conformation of the protein. Due to its position, it has been suggested that this mutation could lead to an unstable intercellular protein complex. In 2003, Suriano et al. identified this germline mutation in two African–American female patients diagnosed with diffuse gastric cancer at 43 years [34]. In this study, functional in vitro analysis of the c.1849G>A mutation in a cell model resulted only in minor functional changes. A recent study identified the same germline variant in 6% (10/165) of African–American patients diagnosed with ductal or mixed carcinoma of the breast [35]. This frequency was similar to the allele frequency identified in the African population in EXAC (0.04622-481/10406, with 15 appearances in homozygous; http://exac.broadinstitute.org/variant/16-68856041-G-A). In the Brazilian population controls, this mutation had allelic frequency of 0.006658. Therefore, due to the mild functional consequences observed in vitro assays and its high allele frequency, especially in the African-descendent population, this variant was classified as likely benign. This classification is in concordance with the majority of the CLINVAR submission in which 14 of the 17 submission classified this variant as benign or likely benign (https://www.ncbi.nlm.nih.gov/clinvar/variation/12232/, accessed November 2018).

### In silico prediction of splice-affecting *CDH1* germline variants

The results of in silico tools for the prediction of splicing defect are described in Table 4. Five variants have been previously described as benign and in silico analyses really indicated their low pathogenicity: c.324A>G, c.345G>A, c.532-18C>T, c.833-16C>G, and c.2439+10C>T. Only one novel variant showed potential to affect the splicing process by three prediction tools: c.387G>T. This missense variant is located in the last nucleotide of the exon 3, leading to an amino acid substitution (glutamine-to-histidine). This variant may cause the alteration of the donor site and has the potential to affect splicing.

**Table 4:** Analysis of *CDH1* germline variants using in silico tools for splicing defect prediction

Exon	Variant	HSF 3.0	MaxEnt	NNSplice	NetGene2	CLINVAR	LOVD
		0–100 (Δ%)	− 20 to 20 (Δ%)	0–1 (Δ%)	0–1 (Δ%)	(No. submission)	
**Intron 1**	c.49-59G>T	**89.94 → 79.07**	**3.46 → 5.91**	=	0.43 **→** 0.44	NA	NA
		**(− 12.09%)**	**(**+ **70.81%)**		(+ 2.3%)		
**Intron 2**	c.163+57	=	NR	=	0.43 **→** 0.44	NA	NA
					(+ 2.3%)		
**Intron 2**	c.163+59	=	NR	=	0.37 **→** 0.36	NA	NA
					(− 2.7%)		
**Exon 3**	c.313T>A	89.8 **→** 85.88	**5.61 → 2.66**	=	0.28 **→** 0.25	NA	NA
	(S105T)	(− 4.37%)	**(**− **52.58%)**		(− 10.71%)		
**Exon 3**	c.324A>G	89.8 **→** 92.92	5.61 → 6.86	=	0.79 **→** 0.82	Benign (2)	NA
	(R108R)	(+ 3.49%)	**(**+ 22.28%)		(+ 3.79%)	Likely benign (1)	
**Exon 3**	c.345G>A	69.5 **→** 69.1	NR	=	0.79 **→** 0.66	Benign (3)	UE
	(T115T)	(− 0.58%)			(− 16.45%)		
**Exon 3**	c.387G>T	**88.42 →59.47**	**8.87 → 0.28**	**0.98 → 0.53**	=	NA	NA
	(Q129H)	**(**− **32.74%)**	**(**− **96.84%)**	**(**− **45.92%)**			
**Intron 3**	c.387+27C>T	=	5.02 **→** 4.94	0.77 **→** 0.83	0.79 **→** 0.76	NA	UE
			(− 1.59%)	(+ 7.79%)	(− 3.79%)		
**Intron 3**	c.388-44C>T	=	NR	=	0.54 **→** 0.56	NA	UE
					(+ 3.7%)		
**Intron 4**	c.532-18C>T	=	10.59 **→** 10.98	=	=	Benign (2)	UE
			(+ 3.68%)			Likely benign (1)	
**Intron 6**	c.833-16C>G	=	8.1 **→** 7.8	0.88 **→** 0.74	0.30 **→** 0.25	Benign (1)	UE
			(− 3.7%)	(− 15.90%)	(− 16.67%)	Likely benign (1)	
**Exon 11**	c.1676G>A	**74.28 → 45.34**	NR	=	0.27 **→** 0.25	NA	NA
	(S559N)	**(− 38.96%)**			(− 7.40%)		
**Exon 12**	c.1806C>A	79.72 **→** 77.14	NR	=	0.25 **→** 0.19	NA	NA
	(F602L)	(− 3.24%)			(− 24.00%)		
**Intron 15**	c.2439+10C>T	67.5 **→** 68.75	NR	=	=	Benign (2)	UE
		(+ 1.85%)				Likely benign (1)	
**Intron 15**	c.2439+56T>G	66.32 **→** 70.62	− 2.35 → 6.14	0.99 **→**0.99	0.92 **→** 0.91	NA	NA
		(+ 6.48%)	**(**+ 361.28%)	0%	(− 1.09%)		

Values displayed on the left side and on the right side of the arrow refer, respectively, to wild-type and mutant alleles

Bold values indicate the significant values for splice site effect, according to splice prediction tools cited in materials and methods

*UE* unknown effect, *like* likely, *NA* not available, *NR* no result

^=^Unchanged between wild and mutant

### *CDH1* germline variants of unknown significance and patients’ characteristics

Based on allele frequency, literature/databases searches, and in silico analysis, four variants were classified as VUS according to the recommendations of the American College of Medical Genetics and Genomics [22] (Table 5). These variants were identified in patients diagnosed with diffuse gastric adenocarcinoma; however, E-cadherin immunoexpression was present in these tumor samples and these patients did not report a gastric cancer family history. These variants had never been described previously. It is noteworthy that VUS carriers were also exposed to environmental risk factors such as *H. pylori* infection, alcoholism, obesity, smoking, and red/processed meat consumption.

**Table 5:** Novel missense *CDH1* germline variants with potential pathogenic effect

Prob	Variant	Sex	Age	HT	CS	Molecular genetic factors					Socio-environmental factors						
						In silico	IHC *CDH1*	FH	AF ExAC	IGCLC 2015	Hp	Tob	Alc	Red meat	Proc meat	Fruits	BMI
GH152	c.313T>A (S105T)	M	27.7	DGA	IV	Damaging	+^a^	No	NA	+	+	Yes	Yes	6–7×/week	6–7×/week	3–5×/week	22.8
GH68	c.387G>T (Q129H)	M	54.4	DGA	IV	Damaging	+^a^	Father PC, 88a	0.0000083	−	NA	Yes	Yes	3–5×/week	1–2×/week	6–7×/week	40.9
GH12	c.1676G>A (S559N)	F	45	DGA	IIIC	Damaging	+^b^	Sister CRC, 33a	NA	−	NA	Yes	Yes	1–2×/week	< 1×/week	1–2×/week	26.6
GH25	c.1806C>A (F602L)	F	38.6	DGA	IV	Damaging	+^a^	GrMo BC, 50a	NA	+	NA	Yes	Yes	3–5×/week	< 1×/week	6–7×/week	31.2

In silico analysis—considered damaging if prediction was non-neutral by at least two in silico tool (Human Splicing Finder, NNS, MaxEntScan, NetGene2, SIFT, Align-GV/GD, MutationTaster, PROVEAN, and Poly-Phen2) have indicated pathogenic potential

*AF* allelic frequency, *Alc* alcohol, *BC* breast cancer, *BMI* body mass index before the diagnosis of cancer, *CRC* colorectal cancer, *DGA* diffuse gastric adenocarcinoma, *EXAC* Exome Aggregation Consortium, *F* female, *FH* family history, *GrMo* grandmother, *Hp Helicobacter pylori, HT* histology, *IGCLC* international gastric cancer linkage consortium criteria, *IHC* immunohistochemistry, M male, *NA* not available, *PC* prostate cancer, *proc* processed, *Tob* tobacco

^a^IHC performed in peritoneal carcinomatosis biopsy

^b^IHC performed in gastric tumor; +, positive

### Evaluation of *CDH1* mRNA from tumor samples

Nine FFPE tumor samples, presenting seven different variants, were further studied to evaluate mRNA splicing effects.

**Fig. 1:**
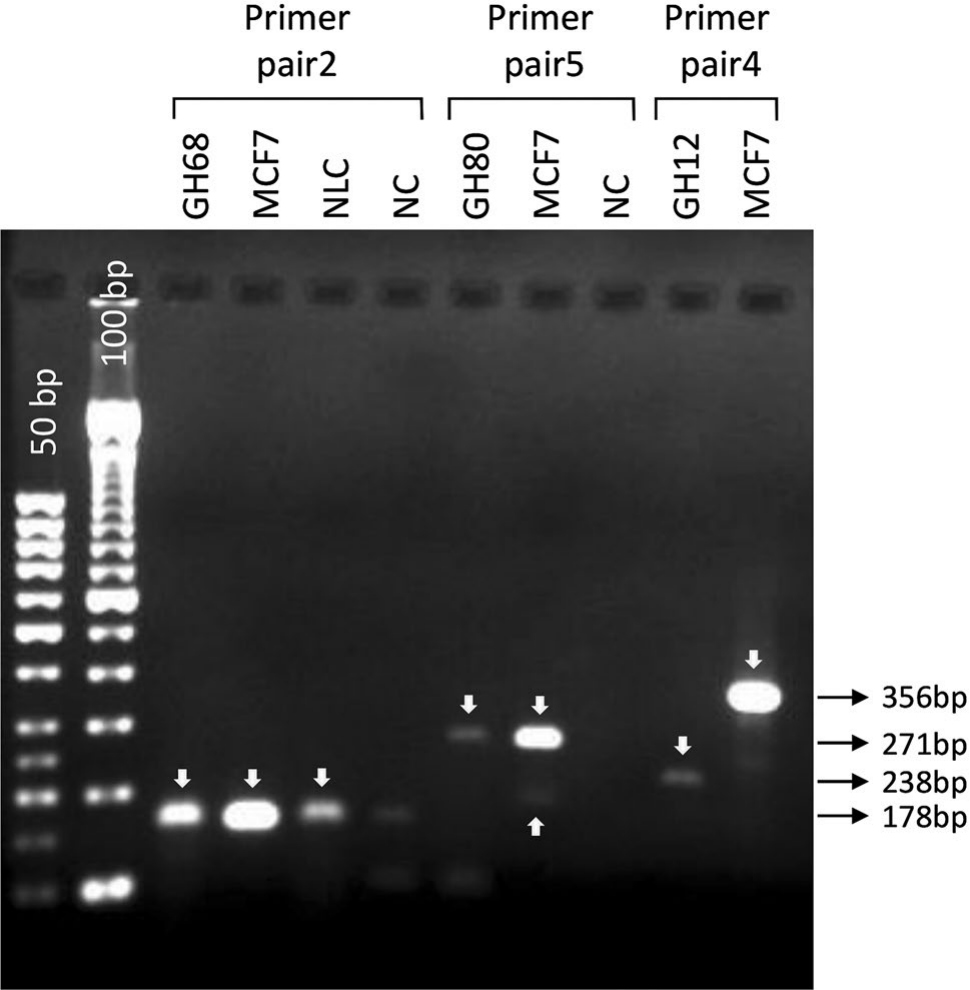
Electrophoresis of amplicons in agarose gel. RNA was extracted from FFPE tumor samples, and after RT-PCR, amplicons detected in agarose gel were cloned in bacteria and sequenced.* NLC* normal breast tissue (FFPE),* NC* negative control,* MCF7* breast cancer cell line,* GH* gastric cancer FFPE samples,* Primer pair 2CDH1* Exons 3–4,* Primer pair 5CDH1* Exons 15–16,* Primer pair 4CDH1* Exons 10–12

After mRNA extraction and cDNA synthesis, electrophoresis in agarose gel revealed  no PCR product for six samples, probably due to mRNA degradation in the FFPE fragment (n=5) or insufficient material (*n* = 1).  However, PCR products were detected for three tumor samples (Fig. 1): an amplicon of 178 bp, representing exons 3–4, on sample GH68 and an amplicon of 271 bp, representing exons 15–16, on sample GH80. There was an unexpected amplicon of 238 bp on sample GH12. These products were cloned for cDNA sequencing

PCR products were also submitted to electrophoresis in polyacrylamide gel: for sample GH68, besides the fragment of 178 bp, another one of approximately 300 bp was detected, and for sample GH80, besides the amplicon of 271 bp, an additional product of 100 bp was detected. Sequencing the products of samples GH12, GH68 (amplicon 300 bp) and GH80 (amplicon 100 bp) revealed only the universal primer M13 sequence. For sample GH68, sequencing of the 178 amplicon showed the wild-type exons 3–4 sequence, without the germline variant c.387G>T, which is located in a canonical splice region, indicating that only the mRNA from the normal allele was recovered (Supplementary Figure 2). In addition, sequencing of the 271 bp amplicon from sample GH80 disclosed the wild-type sequence of exons 15–16.

### Diet and lifestyle habits of EOGC patients

The diet/lifestyle habits of EOGC patients were compared to Brazilian population diet and lifestyle, according to public governmental databases. EOGC patients had a higher red (OR 2.6, 95% CI 1.4–4.9) and processed (OR 3.1, 95% CI 1.6–6.0) meat intake as well as increased fruit consumption compared to eating habits of the Brazilian population (OR 0.4, 95% IC 0.3–0.7). Moreover, a trend to increased consumption of food preserved in salt was observed (OR 1.7, 95% CI 1.0–3.0; *p* = 0.051). However, there were no differences in tobacco and alcohol exposure in the Brazilian population and EOGC patients (Table 6).

**Table 6:** Odds ratio for dietary habits/lifestyle between EOGC patients and Brazilian population

Factors	Exposure	OR	95% CI	*p*	Pop data source
Tobacco	Ever (vs never)	1.5	1.0–2.3	0.080	IBGE, 2013 [29]
Alcohol	Last 6 months (yes vs no)	1.7	0.9–3.0	0.094	INPAD, 2012 [31]
Fruits	< 1x/week (vs ≥ 1x/week)	0.4	0.1–1.2	0.098	SISVAN, 2015 [30]
	≤ 2x/week (vs ≥ 3x/week)	0.7	0.4– 1.1	0.146	
	≤ 5x/week (vs ≥ 6x/week)	0.4	0.3–0.7	< 0.001^a^	
Leaves	< 1x/week (vs ≥ 1x/week)	1.0	0.5–2.1	0.954	SISVAN, 2015 [30]
	≤ 2x/week (vs ≥ 3x/week)	1.1	0.7–1.8	0.584	
	≤ 5x/week (vs ≥ 6x/week)	0.7	0.4–1.1	0.108	
Salty food	≥ 1x/week (vs < 1x/week)	1.0	0.6–1.6	0.991	SISVAN, 2015 [30]
	≥ 3x/week (vs ≤ 2x/week)	1.1	0.7–1.7	0.709	
	≥ 6x/week (vs ≤ 5x/week)	1.7	1.0–3.0	0.051	
Processed meat	≥ 1x/week (vs < 1x/week)	1.0	0.6–1.5	0.827	SISVAN, 2015 [30]
	≥ 3x/week (vs ≤ 2x/week)	1.7	1.0–2.8	0.034^a^	
	≥ 6x/week (vs ≤ 5x/week)	3.1	1.6–6.0	< 0.001^a^	
Red meat	≥ 1x/week (vs < 1x/week)	2.1	0.4–10.9	0.389	IBGE, 2013 [29]
	≥ 3x/week (vs ≤ 2x/week)	1.2	0.6–2.5	0.519	
	≥ 6x/week (vs ≤ 5x/week)	2.6	1.4–4.9	0.003^a^	

*CI* confidence interval, *IBGE* Instituto Brasileiro de Geografia e Estatística, *INPAD* Instituto Nacional de Ciência e Tecnologia  para Políticas Públicas do Álcool e outras Drogas, *OR* odds ratio, *SISVAN* Sistema de Vigilância Alimentar e Nutricional

^a^Statistically significant

## Discussion

Gastric cancer remains one of the leading causes of cancer mortality in developing countries; however, studies evaluating the influence of hereditary factors on gastric cancer burden in these countries are scarce. In a cohort of Brazilian EOGC patients screened for *CDH1* mutations, a total of 24 germline variants were identified, including nine variants never previously described in the literature. Although no definitive pathogenic mutations have been found, four novel missense VUS were detected. The analysis of the socio-environmental risk factors, such as diet and lifestyle habits, revealed that patients with EOGC reported a significantly higher consumption of processed meat and red meat. To our knowledge, the present study represents the largest series analyzing the incidence and spectrum of *CDH1* germline mutations in consecutive and unrelated EOGC patients in Latin America.

HDGC is an autosomal dominant neoplastic syndrome described in 1998 [36, 37]. Male and female *CDH1* mutation carriers have a 70% (95% CI 59–80%) and 56% (95% CI 44–69%) cumulative lifetime risk of developing diffuse gastric cancer, respectively. In addition, the lifetime risk of lobular breast cancer for female carriers is 42% (95% CI 23–68%) [38]. Over the past 2 decades, approximately 160 *CDH1* germline variants have been published; the majority were described in probands with strong family history of cancer and from countries with a low-incidence rate of stomach cancer.

Our study population included predominantly patients diagnosed with diffuse gastric cancer under 40 years old and without family history of cancer. It is important to highlight that probands were originally from all the regions of Brazil. Approximately 50% of the participants were born in Brazilian states outside the Southeast region, with 38.6% coming from Northeast. Therefore, despite the fact that the recruitment was carried out at a single academic center in São Paulo city, the study population was not limited to the inhabitants of this part of Brazil.

Unequivocal pathogenic germline *CDH1* variants were not identified in 88 EOCG patients in Brazil. In a systematic review, that compiled published series usually from regions of low incidence of gastric cancer, only 2.3% of the cases diagnosed with gastric cancer under 35 years carried pathogenic *CDH1 *variants [12]. In high-incidence areas, Corso et al. reported germline variants less frequently, of which 68.8% were missense mutations [39]. Therefore, an absence or a low frequency of definitely pathogenic mutations in our study was already expected, mainly because it was held in a middle/high-incidence country for gastric cancer, where exposure to external risk factors might predominate and, thereby, increase the risk of sporadic cases.

Hansford et al. have recently cataloged all *CDH1* variants identified so far [38]. Among the 155 mutations described, 126 were pathogenic and 29 were VUS. Among the 126 pathogenic mutations, only 16% were missense. On the other hand, among the 29 VUS, 86% were missense. In our study, among the 24 variants identified, 33.3% (8/24) were classified as benign-polymorphisms (4 intronic and 4 synonymous), 20.8% (5/24) as benign (4 intronic and 1 synonyms), 29.2% (7/24) as probably benign (5 intronic, 1 synonymous and 1 missense), and 16.7% (4/24) as VUS (4 missense). Despite the fact that 16.7% of mutations found in *CDH1* are novel and have low allele frequency, the ideal approach that can definitely assess the potential pathogenicity of these changes is still a matter of debate [7, 40–42].

Among the four VUS, c.387G>T presented a low allele frequency (allele 1/119896; http://exac.broadinstitute.org/variant/16-68835796-G-T) and the other 3 variants were not described in EXAC. Therefore, the classification of the pathogenicity of these variants might be possible only by studying the other families with the same variants and with family history suggestive of hereditary diffuse gastric cancer syndrome or performing functional tests. All carriers identified in our study were diagnosed with the early onset diffuse gastric adenocarcinoma. However, they did not report family history of stomach and/or breast cancer. Thus, segregation studies were not a viable approach. In addition, these variants have never been previously published or reported in CLINVAR and/or LOVD. Computational algorithms were used to predict their pathogenicity, but the results were discordant among the prediction tools. These findings highlight that in silico predictions should be used with caution, as a complementary tool, and that important clinical decisions regarding the interpretation of variants cannot be made based on the in silico outcomes alone [42, 43]. Functional impact on splicing experiments, which may help in the characterization of newly identified VUS, were performed.

The variant c.387G>T was further tested in mRNA from the patient’s tumor sample, because it is located on the exon/intron boundary (last base exon 3). The splicing prediction tool indicated that the splicing site might be lost and a probable novel splicing site might be located approximately 1460 bases inside the intron (NNsplice: available at https://omictools.com/nnsplice-tool, accessed November 2018). Our results, however, detected only the mRNA transcribed from the normal allele, because even the variant was not present in the amplicon. Based on these results, we still cannot infer the pathogenicity of the missense variant.

Our study was limited to explore the presence of germline variants only in the *CDH1* gene and not in other gastric cancer predisposing genes. Although *CDH1* is the most relevant gene, explaining about 40% of the cases, other genes may be involved in familial gastric cancer. Recently, new candidates have been identified including *CTNNA1*, *BRCA1*, *BRCA2*, *STK11*, *PRSS1*, *PALB2*,* ATM*, *MSR1*, *SDHB*, *RAD51*, and *MAP3K6* [38, 44–46], but the clinical relevance of these findings still requires further validation.

Although the intestinal type of gastric cancer is associated with diet and lifestyle habits, the contribution of known modifiable risk factors to the incidence of diffuse-type gastric cancer is still under investigation. In the report of Continuous Update Project, processed meat intake and alcohol consumption above moderate levels were associated with the increased risk of gastric cancer regardless of histology subtype. In addition, there is limited evidence if the consumption of grilled fish, meat, and fruit affects the risk of developing gastric cancer [47]. In our study, in which the diffuse type was predominant, patients with EOGC reported a significantly higher consumption of red and processed meat, as well as fruits, compared to the eating habits of the Brazilian population. We did not find an association between alcohol intake and gastric cancer; however, alcohol exposure was measured in a very distinctive manner, evaluating any exposure in the last 6 months and not taking into account the amount of daily alcohol consumption (in grams per day) as usually reported [48]. Interestingly, although reports from Europe suggest that consuming little or no fruit increases the risk of gastric cancer [49], our findings showed the opposite effect and, as a consequence, require further investigation. The information about *H. pylori* infection is missing for the majority of our patients, but it is important to acknowledge that this well-established risk factor may also contribute to diffuse gastric cancer risk. Indeed, gastric cancer risk likely reflects a complex interaction among various diet and lifestyle habits, and *H. pylori* infection may function as a confounder or potential effect modifier [50].

In conclusion, unequivocal pathogenic germline *CDH1* variants did not contribute significantly for EOGC predisposition in our cohort and the assessment of the potential pathogenicity of missense variants still represents a major challenge. In addition, it was observed that the nutrition habits of our patients are inadequate. For neoplasms like gastric cancer, in which the influence of external factors such as diet might increase the risk the disease, this information is relevant and warrants further investigation for the purpose of health promotion in the Brazilian population.

## Electronic supplementary material

Below is the link to the electronic supplementary material.

Supplementary material 1 (DOC 2067 KB)
