# Supplementary material for: Frequency of CDH1 germline variants and contribution of dietary habits in early age onset gastric cancer patients in Brazil
Source: Gastric Cancer. 2019 Mar 20;22(5):920–31. doi: 10.1007/s10120-019-00945-9 (PMC6694034; doi:10.1007/s10120-019-00945-9)
Supplement: Supplementary file 1 — Supplementary material 1 (DOC 2067 KB) [file 10120_2019_945_MOESM1_ESM.doc]

**Supplementary Materials**

***Polymerase chain reaction (PCR) Amplification and Sanger Sequencing***

The complete coding regions of *CDH1* (GenBank, NM_004360.4), including 50-100 base pairs (bp) of non-coding sequences, flanking the 5’ and 3’ ends of each exon, were amplified by PCR using 15 pairs of primers for *CDH1* gene (Supplementary Table 1). AmpliTaq Gold enzyme 250U

(final concentration 0.4U/µl) (Applied Biosystems, Foster City, CA, USA), 1X AmpliTaq Gold buffer, 1.5-3.0 mM AmpliTaq Gold MgCl_2_, 0.16 mM dNTP (Applied Biosystems, Foster City, CA, USA), 0.4 mM of each primer and 50 ng of DNA in a total volume of 10 µL in a GeneAmp 9700 Thermal Cycler (Applied Biosystems, Foster City, CA, USA). The PCR cycling consisted of 1 cycle at 95°C for 10 min, 35 cycles at 94°C for 30 sec, 50-70°C for 40 sec, 72°C for 50 sec, and 1 cycle at 72°C for 7 min. PCR products were loaded onto a 1.5% agarose gel, stained with GelRed Nucleic Acid Stain (Biotium, Hayward, CA, USA), and evaluated. Each PCR product was then treated with 1 µL of illustra™ ExoStar™ 1-Step (GE Healthcare Bio-Sciences, Pittsburgh, PA, USA) and incubated at 37°C for 15 minutes followed by 80°C for 15 minutes. All PCR products were sequenced in both forward and reverse directions using BigDye® Terminator v3.1 (Life Technologies or Applied Biosystems) according to the manufacturer’s instructions, and were analyzed on a 3500 Genetic Analyzer (Applied Biosystems - Hitachi). Sequences obtained were visualized by Chromas v2.33 (Technelysium Pty. Ltd.) and by Mutation Surveyor v3.20 (SoftGenetics LLC).

| **Supplementary Table 1 – Oligonucleotides for *CDH1* gene sequencing. F, forward; R, reverse** | | | | | |
| --- | --- | --- | --- | --- | --- |
| ***CDH1* Exon** |  | **Primer (5’-3’)** | **Length (bp)** | **MgCl_2_ (mM)** | **°C annealing** |
| **Exon 1** | F  R | TACGGGGGGCGGTGCCTCCGGGG  CCGCAAGCTCCGCGCCCCAG | 282 | 2 mM^a^ | 70°C |
| **Exon 2** | F  R | TGTTGGTTTCGGTGAGCAG  GGTGTGGGAGTGCAATTTCT | 261 | 2 mM | 54°C |
| **Exon 3** | F  R | CGCTCTTTGGAGAAGGAATG AACGGTACCAAGGCTGAGAA | 376 | 2 mM | 55°C |
| **Exon4-5** | F  R | TGTACACTGCCCACAGAAGG  GTTACCCCGGTGTCAACAAG | 574 | 2 mM | 55.8°C |
| **Exon 6** | F  R | GCCCCTTCTCCCATGTTT CTTTGGGCTTGGACAACACT | 287 | 3 mM | 60°C |
| **Exon 7** | F  R | GGGCAGAATTGGATTAAGCA  TGTCCACGGGATTGAGCTA | 320 | 2 mM | 53°C |
| **Exon 8** | F  R | CTGGGCTAGGCCAAAGGT  CCATGAGCAGTGGTGACACTT | 272 | 2 mM | 55.8°C |
| **Exon 9** | F  R | AATCCTTTAGCCCCCTGAGA  AGGGGACAAGGGTATGAACA | 326 | 2 mM | 54°C |
| **Exon 10** | F  R | CCAAAAGCAACAGTTAAGGA  CAAATGACAAAATGCCATGA | 433 | 2 mM | 50°C |
| **Exon 11** | F  R | AGCGCTTAAGCCGTTTTCA  GAGGGGCAAGGAACTGAACT | 283 | 2 mM | 54°C |
| **Exon 12** | F  R | AAGGCAATGGGGATTCATTA  ATTGAAAGGTGGGGATCTGG | 363 | 3 mM | 60°C |
| **Exon 13** | F  R | CAATTTTATTCTGGAATGAGCTTTT  CAGGAAATAAACCTCCTCCATTT | 363 | 2 mM | 50°C |
| **Exon 14** | F  R | GCTGCTTCTGGCCTTCTTTA  GCTGTTTCAAATGCCTACCTCT | 281 | 2 mM | 55°C |
| **Exon 15** | F  R | TGAACATAGCCCTGTGTGTATG  TTTTTGACACAACTCCTCCTG | 281 | 2 mM | 53°C |
| **Exon 16** | F  R | AGACTTCTTGCCCCAGATGA AACCACCAGCAACGTGATTT | 347 | 2 mM | 54°C |
| Symbol: ^a^, add of DMSO 5%. | | | | | |

**Supplementary Table 2 – Clinical-pathological characteristics, family history and germline variants of EOGC (n=88)**

| ID | Age | CS | Hist | Grade | FH+ GC | FH+ BC | Variants | | | | | | | |
| --- | --- | --- | --- | --- | --- | --- | --- | --- | --- | --- | --- | --- | --- | --- |
| GH-01-01 | 32.9 | IB | other | poor | no | no | 163+57G>A | 2076T>C |  |  |  |  |  |  |
| GH-02-01 | 40.8 | IA | dif | poor | no | no | 48+6C>T | 1937-13T>C | 2076T>C |  |  |  |  |  |
| GH-04-01 | 35.9 | IIIA | dif | poor | yes | no | 48+6C>T | 163+57G>A | 1849G>A | 2076T>C |  |  |  |  |
| GH-07-01 | 31 | IA | dif | poor | yes | no | 48+6C>T | 2076T>C |  |  |  |  |  |  |
| GH-12-01 | 45 | IIIC | dif | poor | no | no | 1676G>A | 2076T>C |  |  |  |  |  |  |
| GH-14-01 | 40.5 | IIIC | dif | poor | no | no | 48+6C>T | 49-59G>T | 163+57G>A | 163+59G>C | 388-44G>A | 1937-13T>C | 2076T>C | 2164+16insA |
| GH-15-01 | 36 | IIIC | dif | poor | no | no | 48+6C>T | 163+57G>A | 163+59G>C | 2076T>C |  |  |  |  |
| GH-16-01 | 42.4 | IV | dif | poor | no | no | 48+6C>T | 163+57G>A | 163+59G>C | 2076T>C |  |  |  |  |
| GH-17-01 | 37.2 | IIB | dif | poor | no | no | 48+6C>T | 2076T>C | 2164+16insA | 2253C>T |  |  |  |  |
| GH-18-01 | 31 | IIIA | dif | poor | no | no | 48+6C>T | 833-16C>G | 2076T>C |  |  |  |  |  |
| GH-19-01 | 39.7 | IV | other | poor | no | no | 48+6C>T | 2076T>C |  |  |  |  |  |  |
| GH-20-01 | 49.7 | IA | other | poor | yes | no | 48+6C>T | 2076T>C | 2439+10C>T |  |  |  |  |  |
| GH-21-01 | 31.7 | IIA | dif | NA | no | no | 48+6C>T | 2076T>C |  |  |  |  |  |  |
| GH-23-01 | 31 | IA | dif | NA | no | no | 48+6C>T |  |  |  |  |  |  |  |
| GH-24-01 | 32.5 | IIA | dif | poor | no | no | 48+6C>T | 2076T>C |  |  |  |  |  |  |
| GH-25-01 | 38.6 | IV | dif | poor | no | yes | 48+6C>T | 1806C>A | 2076T>C |  |  |  |  |  |
| GH-26-01 | 33.3 | IV | intest | mod | yes | no | 48+6C>T | 2076T>C |  |  |  |  |  |  |
| GH-27-01 | 40.8 | IV | intest | mod | no | no | 48+6C>T | 2076T>C | 2164+16insA | 2253C>T |  |  |  |  |
| GH-28-01 | 33.6 | IV | dif | poor | no | yes | 1896C>T | 2076T>C | 2634C>T |  |  |  |  |  |
| GH-30-01 | 55 | IIA | dif | poor | no | no | 49-59G>T | 163+57G>A | 2076T>C |  |  |  |  |  |
| GH-31-01 | 43.3 | IV | dif | poor | no | no | 48+6C>T | 1849G>A | 2076T>C |  |  |  |  |  |
| GH-32-01 | 48 | IIB | dif | poor | no | no | 48+6C>T | 1849G>A | 2076T>C |  |  |  |  |  |
| GH-33-01 | 50.1 | IA | dif | poor | yes | yes | 2076T>C |  |  |  |  |  |  |  |
| GH-34-01 | 27.5 | IV | dif | poor | no | no | 48+6C>T | 2076T>C |  |  |  |  |  |  |
| GH-35-01 | 38 | IV | dif | poor | no | no | 48+6C>T | 2076T>C |  |  |  |  |  |  |
| GH-36-01 | 46.9 | IIB | mixed | NA | no | no | 48+6C>T |  |  |  |  |  |  |  |
| GH-37-01 | 25.3 | IV | other | poor | no | no | 48+6C>T | 2076T>C |  |  |  |  |  |  |
| GH-38-01 | 32.7 | IIIA | dif | NA | no | no | 2076T>C |  |  |  |  |  |  |  |
| GH-39-01 | 28.,4 | IV | dif | poor | yes | no | 48+6C>T | 833-16C>G | 1896C>T | 2076T>C |  |  |  |  |
| GH-40-01 | 48.2 | IIIC | dif | poor | no | yes | 48+6C>T | 2076T>C |  |  |  |  |  |  |
| GH-42-01 | 30.2 | IIB | dif | poor | no | no | 48+6C>T | 2076T>C |  |  |  |  |  |  |
| GH-43-01 | 47.6 | IV | dif | poor | no | no | 48+6C>T | 2076T>C |  |  |  |  |  |  |
| GH-44-01 | 25.8 | IV | dif | NA | no | no | 48+6C>T | 2076T>C |  |  |  |  |  |  |
| GH-45-01 | 21.2 | ND | dif | poor | no | no | 163+57G>A | 163+59G>C | 1937-13T>C | 2076T>C |  |  |  |  |
| GH-46-01 | 34.7 | IIIC | dif | poor | no | no | 48+6C>T | 531+10G>C | 2076T>C |  |  |  |  |  |
| GH-47-01 | 37.5 | IIIA | dif | NA | yes | no | 48+6C>T | 2076T>C |  |  |  |  |  |  |
| GH-48-01 | 42.4 | IIIC | dif | poor | yes | no | 2076T>C | 2164+16insA | 2253C>T |  |  |  |  |  |
| GH-49-01 | 42.5 | IB | dif | poor | no | yes | 48+6C>T | 2076T>C | 2164+16insA | 2253C>T |  |  |  |  |
| GH-50-01 | 42.2 | IIIA | dif | poor | no | no | 48+6C>T | 2076T>C |  |  |  |  |  |  |
| GH-51-01 | 44.1 | IA | dif | NA | yes | no | 48+6C>T | 1896C>T | 2076T>C | 2634C>T |  |  |  |  |
| GH-52-01 | 48.5 | IIB | dif | poor | yes | no | 48+6C>T | 2076T>C |  |  |  |  |  |  |
| GH-53-01 | 38.,5 | IIIB | dif | poor | no | no | 48+6C>T | 49-59G>T | 1937-13T>C | 2076T>C | 2439+56T>G |  |  |  |
| GH-54-01 | 34.6 | IIA | mixed | poor | no | no | 48+6C>T | 49-59G>T | 2076T>C | 2439+56T>G |  |  |  |  |
| GH-55-01 | 42.7 | IIB | dif | well | no | no | 48+6C>T | 387+27C>T | 2076T>C |  |  |  |  |  |
| GH-56-01 | 31.6 | IV | dif | poor | yes | yes | 48+6C>T | 1849G>A | 1937-13T>C | 2076T>C |  |  |  |  |
| GH-58-01 | 28.,2 | IA | dif | poor | no | no | 48+6C>T | 2076T>C |  |  |  |  |  |  |
| GH-59-01 | 34.3 | IIIB | mixed | poor | yes | no | 48+6C>T | 2076T>C |  |  |  |  |  |  |
| GH-60-01 | 36.2 | IV | dif | poor | no | no | 48+6C>T |  |  |  |  |  |  |  |
| GH-61-01 | 44.7 | IB | mixed | poor | yes | no | 48+6C>T | 2076T>C |  |  |  |  |  |  |
| GH-62-01 | 36.,4 | IV | dif | poor | no | no | 48+6C>T | 49-59G>T |  |  |  |  |  |  |
| GH-63-01 | 51.6 | IIIB | dif | poor | no | no | 48+6C>T | 2076T>C |  |  |  |  |  |  |
| GH-64-01 | 38.,9 | IV | dif | poor | no | no | 48+6C>T | 1937-13T>C | 2076T>C |  |  |  |  |  |
| GH-65-01 | 28.3 | IV | dif | poor | no | no | 48+6C>T | 531+10G>C | 2076T>C |  |  |  |  |  |
| GH-67-01 | 55.6 | IIIC | dif | poor | no | no | 48+6C>T | 345G>A | 2076T>C |  |  |  |  |  |
| GH-68-01 | 54.4 | IV | dif | poor | no | no | 48+6C>T | 387G>T |  |  |  |  |  |  |
| GH-70-01 | 40.7 | IIIA | other | mod | no | no | 48+6C>T |  |  |  |  |  |  |  |
| GH-72-01 | 51.,5 | IIB | dif | poor | no | no | 48+6C>T | 324G>A | 1896C>T | 2076T>C | 2634C>T |  |  |  |
| GH-73-01 | 55.9 | IV | dif | NA | no | no | 48+6C>T | 2076T>C |  |  |  |  |  |  |
| GH-74-01 | 36.7 | IV | dif | poor | no | no | 48+6C>T | 2076T>C | 2253C>T |  |  |  |  |  |
| GH-75-01 | 37.2 | IIB | other | mod | no | no | 48+6C>T | 1896C>T | 2076T>C |  |  |  |  |  |
| GH-76-01 | 38.9 | IIIB | dif | poor | no | no | 48+6C>T | 49-59G>T | 163+57G>A | 2076T>C |  |  |  |  |
| GH-77-01 | 45.2 | IV | other | NA | yes | no | 48+6C>T | 163+57G>A |  |  |  |  |  |  |
| GH-78-01 | 31.5 | IV | dif | poor | no | no | 48+6C>T | 1937-13T>C | 2076T>C |  |  |  |  |  |
| GH-79-01 | 41.5 | IV | dif | poor | no | no | 48+6C>T | 1937-13T>C | 2076T>C |  |  |  |  |  |
| GH-80-01 | 36.4 | IIIB | intest | poor | no | no | 48+6C>T | 163+57G>A | 2439+56T>G |  |  |  |  |  |
| GH-81-01 | 39.4 | IIIA | dif | poor | yes | yes | 48+6C>T | 49-59G>T | 163+57G>A | 1896C>T | 2076T>C | 2634C>T |  |  |
| GH-85-01 | 42.8 | IIIB | other | poor | no | no | 48+6C>T | 1937-13T>C | 2076T>C |  |  |  |  |  |
| S-GAST-101 | 41.1 | IIIC | dif | poor | no | no | 48+6C>T | 2076T>C |  |  |  |  |  |  |
| S-GAST-108 | 53.4 | IIA | intest | poor | no | no | 48+6C>T | 2076T>C |  |  |  |  |  |  |
| S-GAST-109 | 41.6 | IB | dif | poor | no | no | 48+6C>T | 532-18C>T | 1937-13T>C | 2076T>C |  |  |  |  |
| S-GAST-114 | 55.5 | IA | mixed | poor | no | no | 48+6C>T |  |  |  |  |  |  |  |
| S-GAST-116 | 50 | IA | dif | poor | no | no | 2076T>C |  |  |  |  |  |  |  |
| S-GAST-124 | 47.5 | IIIB | dif | poor | no | no | 48+6C>T | 1937-13T>C | 2076T>C | 2253C>T |  |  |  |  |
| S-GAST-135 | 48.5 | IIB | mixed | poor | no | no | 531+10G>C | 2076T>C |  |  |  |  |  |  |
| S-GAST-143 | 45.8 | IA | other | poor | no | no | 48+6C>T | 2076T>C |  |  |  |  |  |  |
| S-GAST-144 | 35.4 | IV | dif | poor | yes | no | 48+6C>T | 2076T>C |  |  |  |  |  |  |
| S-GAST-145 | 37.6 | IV | dif | poor | no | no | 48+6C>T | 2076T>C |  |  |  |  |  |  |
| S-GAST-146 | 39.6 | IIIB | dif | poor | no | no | 48+6C>T | 163+57G>A | 2076T>C |  |  |  |  |  |
| S-GAST-147 | 36,5 | IV | dif | NA | no | no | 48+6C>T | 163+57G>A | 531+10G>C | 2076T>C |  |  |  |  |
| S-GAST-148 | 39.2 | IV | other | poor | no | no | 48+6C>T | 49-59G>T | 163+57G>A | 2076T>C | 2253C>T |  |  |  |
| S-GAST-149 | 37.4 | IV | dif | poor | no | no | 48+6C>T | 163+57G>A | 2076T>C |  |  |  |  |  |
| S-GAST-150 | 38.2 | IV | dif | NA | no | no | 48+6C>T | 2076T>C |  |  |  |  |  |  |
| S-GAST-151 | 23.8 | IV | other | NA | yes | no | 48+6C>T | 49-59G>T | 163+57G>A |  |  |  |  |  |
| S-GAST-152 | 27.7 | IV | other | poor | no | no | 48+6C>T | 163+57G>A | 163+59G>C | 313T>A | 2076T>C |  |  |  |
| S-GAST-153 | 39.7 | IV | dif | poor | no | no | 48+6C>T | 1937-13T>C | 2076T>C |  |  |  |  |  |
| S-GAST-154 | 22.8 | IV | dif | poor | no | no | 48+6C>T | 1937-13T>C | 2076T>C |  |  |  |  |  |
| S-GAST-155 | 36.7 | IIIA | dif | poor | yes | no | 48+6C>T | 2076T>C | 2164+16insA | 2253C>T |  |  |  |  |
| S-GAST-156 | 20 | IV | other | NA | no | no | 48+6C>T | 2076T>C |  |  |  |  |  |  |

Abbreviations: CS, clinical stage; dif, diffuse; hist, histology; FH+ BC, 1st or 2nd degree relative diagnosed with breast cancer; FH+ GC, 1st or 2nd degree relative diagnosed with gastric cancer; intest, intestinal; mod, moderately; NA, not available

***Multiplex Ligation-Dependent Probe Amplification (MLPA) of CDH1 Gene***

All patients were investigated for large rearrangements, specifically deletions and duplications, using the Multiplex ligation-dependent probe amplification (MLPA) commercial kit: *SALSA® MLPA® P083-050R probe mix* (MRC-Holland, Amsterdam, The Netherlands).

At first, 80 ng of genomic DNA in 2.5 µl of ultrapure water was denatured for fifteen minutes at 98°C, after which 1.5 μl of the probe mixture (0.75 µl of MLPA probe and 0.75 µl of MLPA buffer) were added. The DNA sample and probe mixture were heated at 95°C for 1 minute and incubated at 60°C overnight (19 h). Afterwards, ligation was performed by adding 16 µl of ligase mix (1.5 µl of ligase buffer A, 1.5 µl of ligase buffer B, 0.5 µl of Ligase-65 and 12.5 µl of water) and maintaining the temperature at 54°C for 15 minutes. Ligase was inactivated by incubation for five minutes at 98°C. Amplification was performed through addition of 5 µl of Polymerase mix (1 µl of Salsa PCR primers, 0.25 µl of Salsa Polymerase and 3.75 µl of water) and heating at 95°C for 1 minute. PCR was carried out for 35 cycles (30 sec at 95°C, 30 sec at 60°C and 60 sec at 72°C) followed by 20 minutes at 72°C in a Veriti 96-well Thermal Cycler (Applied Biosystems). Afterwards, 1 µl of PCR product diluted 1:10 in water, 0.075 µl of GeneScan™ 600 LIZ® dye Size Standard v2.0 (*Applied Biosystems*, Foster City, CA, USA - 4408399) and 9 µl of HiDi Formamide (Thermo Fisher Scientific, Waltham, MA USA - [4440753](https://www.thermofisher.com/order/catalog/product/4440753)) were mixed and incubated at 80°C for 2 minutes in a Veriti 96 well Thermal Cycler (Applied Biosystems, Foster City, CA, USA). For normalizing the data, at least three genomic DNA samples obtained from peripheral blood cells of healthy donors were always run as controls in each analysis. The fragments were analyzed on an Applied Biosystems 3500 Genetic Analyzer (Applied Biosystems - Hitachi) and the resulting data was analyzed through Coffalyser.NET software (MRC Holland, Amsterdam, Netherlands). Normal values were considered when ratio was between 0.7 and 1.3.

**RNA extraction from paraffin-embedded samples (FFPE)**

Samples harboring *CDH1* variants of interest were chosen for RNA extraction, further cloning and sequencing, as shown in the Supplementary table 3.

Approximately five FFPE sections of 10 to 20 µm were obtained for each tumor sample, totalizing around 80 μm of tissue for each. RNA was extracted using RecoverAll™ Total Nucleic Acid Isolation Kit (Ambion® Inc, Austin, Texas, USA). For deparaffinization, 1 ml of Xylene was added to the samples, and incubated at 50°C for 3 minutes. Afterwards, the microtubes were centrifuged and xylene was discarded. Samples were washed twice with Ethanol 100% (1ml) to remove residual xylene. Ethanol was separated from pellet through centrifugation at maximum speed for 2 minutes. Supernatant was discarded and the pellet was dried at 40°C for 20 minutes. The digestion phase proceeded by adding 400 µL of Digestion Buffer and 4 µL of Protease. The solution was mixed and incubated at 50°C for 3 hours, followed by 20 minutes at 70°C. After incubation, 480 µL of Isolation Additive solution was added to each sample, followed by 1,100 µL of Ethanol 100%. Sample/solution mixture passed through a filter cartridge by centrifugation. Mixture was washed with Wash 1 (700 µL), centrifuged at 10,000 xG for 30 seconds. Supernatant was discarded and 500 µL of Wash 2/3 was added. Centrifugation step was repeated, supernatant discarded, and another 30 seconds spin was performed to remove residual fluids from the membrane.

Following addition of 60 µL of DNAse mix (6 µL of 10x DNAse Buffer, 4 µL of DNAse and 50 µL of Nuclease-free water) to the center of the membrane, it was incubated at 25°C for 30 minutes. The wash step with Wash 1 (700 µL) and Wash 2/3 (500 µL) were repeated, the later, twice. Samples placed at the filter cartridge were centrifuged for 1 minute at 10,000 xG to remove residual fluid. Filter cartridge was placed into a new collection tube and the samples were eluted by addition of 30 µL of RNAse-free water, previously heated at 95°C, followed by 5 minutes of incubation at room temperature. Samples were centrifuged at maximum speed for 1 minute and columns were discarded. Sample concentration was verified by Nanodrop spectrophotometer. Afterwards, samples were stored at -80°C.

Supplementary Table 3. mRNA quantification.

| **Samples** | **Variants** | **RNA concentration (ng/ µL)** | **Optical Density values (260/280nm ratio)** |
| --- | --- | --- | --- |
| GH-01 | c.163+57G>A | 725 | 2.0 |
| GH-04 | c.163+57G>A | 152 | 1.9 |
| GH-18 | c.833-16C>G | 81 | 1.9 |
| GH-30 | c.49-59G>T | 92 | 2.0 |
| GH-45 | c.163+57G>A c.163+59G>C | 34 | 1.7 |
| GH-62 | c.49-59G>T | 58 | 2.0 |
| GH-68 | c.387G>T | 63 | 1.9 |
| GH-80 | c.2439+56T>G c.163+57G>A | 68 | 2.0 |
| GH-12 | c.1676G>A | 26 |  |
| S-GAST-146 | c.163+57G>A | 145 | 1.9 |
| S-GAST-151 | c.49-59G>T c.163+57G>A | 108 | 2.0 |
| Normal Breast Tissue (NB) | - | 136 | 1.9 |
| Normal Breast Tissue (NB) | - | 32 | 2.0 |
| MCF-7 | - | 1800 | 2.0 |

**cDNA synthesis with Random Primer**

cDNA was synthesized from 10 pg to 5µg of total RNA, according to the concentration of each sample. For that, we added 11 µL of RNA, 2 µL of Random Primers (0.1 µg/µL), and 1 µL of dNTPS (10 mM) for each reaction. Samples were incubated at 65°C for 5 minutes, followed by one minute of incubation on ice. We then added 6 µL of SuperScript™ III First-Strand Synthesis System mix (4 µL of 5x first strand buffer, 1µL of 0.1 M DTT, and 1 µL of Super Script III RT (200 U/µL). After spinning and mixing by vortex, samples were incubated at 25°C for 5 minutes at 50°C for 55 minutes and at 70°C for 15 minutes.

**RT-PCR (Reverse Transcription- Polymerase Chain Reaction)**

The *CDH1* (GenBank, NM_004360.4) regions of interest were amplified through RT-PCR using five pairs of primers (Supplementary Table 4). We added 0.30 µL of Taq Platinum enzyme (Applied Biosystems, Foster City, CA, USA), 2 µL of 10x buffer, 0.8 µL of MgCl_2_ (50 mM), 0.32µL of dNTP (10mM), 0.4 µL of each primer and 50 ng of cDNA in a total volume reaction of 20 µL (Applied Biosystems, Foster City, CA, USA) in a Veriti Thermal 96-well Cycler (Applied Biosystems, Foster City, CA, USA).The PCR cycling consisted of 1 cycle at 95°C for 5 minutes, 40 cycles at 95°C for 1 minute, 58-60°C for 1 minute, 72°C for 1 minute and 1 cycle at 72°C for 5 minutes. PCR primers were custom designed by IDT primer design tool (Supplementary table 4). PCR products were loaded onto a 1.5% agarose gel, stained with SYBR Safe Nucleic Acid Stain (Biotium, Hayward, CA, USA).

Supplementary table 4. CDH1 primer sequences

| **Primer ID** | ***CDH1***  **Exon** |  | **Primer pair** | **Length (bp)** | **°C annealing** | **Variants investigated**  **(sample ID)** |
| --- | --- | --- | --- | --- | --- | --- |
| Pair 1 | Exon 2  Exon 4 | F  R | GGGCCCTTGGAGCCGCAGCC  TTATTCCTCCCATCAGCTGC | 487 | 60°C | c.49-59G>T  (GH-01, GH30, GH62, S-GAST151)  c.163+57G>A  (GH-04, GH-45, S-GAST-146)  c.163+59G>C  (GH-45) |
| Pair 2 | Exon 3 Exon 4 | F  R | CCACAGATCCATTTCTTGGTC  AGGAGTTGGGTTTTGTGAGC | 178 | 58°C | c.387G>T (GH68) |
| Pair 3 | Exon 6  Exon 8 | F  R | TCTCTCACGCTGTGTCATCC  CAGCTGTTGCTGTTGTGCTT | 393 | 58°C | c.833-16C>G(GH-18) |
| Pair 4 | Exon 10  Exon 12 | F  R | CCAGGAGCCAGACACATTTAT CTGCTGTGAAGGGAGATGTATT | 356 | 58°C | c.1676G>A(GH-12) |
| Pair 5 | Exon 15  Exon 16 | F  R | CGTTGCACCAACCCTCAT  GTACATGTCAGCCAGCTTCTT | 271 | 58°C | c.2439+56T>G  (GH-80) |

After cDNA fragments (5µL) were separated by electrophoresis in a 1.5% agarose gel or in a 6% polyacrylamide gel, in order to identify different fragment sizes, possibly representing the different mRNA variants. cDNA fragments with different sizes were collected from the gel for further purification.

**Polyacrylamide Gel Band Purification Protocol**

Each cDNA fragment was then placed in a 2 mL microtube and 100 µL of RNAse/DNAse free-water was added, incubated at room temperature for 10 minutes and at 95°C for 15 minutes. Samples were mixed by vortex for 5 seconds and spun at 13,000 xG for 2 minutes. Supernatant was transferred to a new tube and 20µL of sodium acetate 3M pH5.4 was added. cDNA samples were precipitated in 2.5 volume of Ethanol 100%, and incubated at 20°C overnight. Next, samples were again centrifuged 12,000xG for 15 minutes. Pellets were washed with 1 mL of Ethanol 80%, and centrifuged once more 12,000xG for 5 minutes. The pellets were dried in absorbent paper and dissolved in 30µL of Ultrapure water. Bands purified from Polyacrylamide gel (3 µL) were submitted to amplification by polymerase chain reaction (conditions and primers described at supplementary table 4) and then separated by 1.5% agarose gel electrophoresis.

The cDNA fragments obtained in this step were then purified, from agarose gel, by using the kit Ilustra™ GFX PCR DNA and Gel Band Purification Kit (GE Healthcare Bio-Sciences, Pittsburgh, PA, USA) and cloned into pCR®4-TOPO® Vector by using TOPO TA cloning® kit for sequencing (Invitrogen, Carlsbad, CA, USA).

**TOPO® Vector Cloning Reaction**

**Ligation Reaction**

Purified PCR products from agarose gel (4 µL), 1 µL of salt solution and 1 µL of TOPO® Vector were incubated at 23°C for 10 minutes.

**Transformation pCR®4-TOPO® Vector into competent E-col**i.

Ligation product (4 µL) and competent E. coli (HB101) bacteria (50 µL) were incubated on ice for 30 minutes. Afterwards, we performed a heat-shock phase by placing the reaction at 42°C for 30 seconds, followed by 2 minutes on ice. SOC nutrient media (250 µL) was added, reaction was incubated at 37°C in incubator shaker for 1 hour, followed by a centrifugation at 2,000 xG for 5 minutes. Approximately 150 µL of supernatant were discarded. The remaining 100 µL was plated into LB ampicillin agar petri dishes for each specific sample band, and incubated at 37°C until colonies had grown enough to be picked. After colonies had grown, five different colonies were chosen for each cDNA fragment and placed into independent wells containing LB ampicillin media, which was then incubated in a shaker incubator at 37°C for 2 hours. Next, 3 µL of colony culture were added to a PCR reaction, in order to amplify these clones products before sequencing reaction. M13 universal primers provided by TOPO cloning kit were used for vector+insert amplification. Electrophoresis in 2% agarose gel was performed with 5µL of PCR product to select which clones would be sequenced. Selected clones were then sequenced as shown previously in Sanger sequencing reaction.

**Supplementary Figure 1 – Brazilian regions according to patients place of birth. Regions of Brazil: Centro-Oeste, Central-West; Norte, North; Nordeste, Northeast; Sudeste, Southeast; and Sul, South.**

**
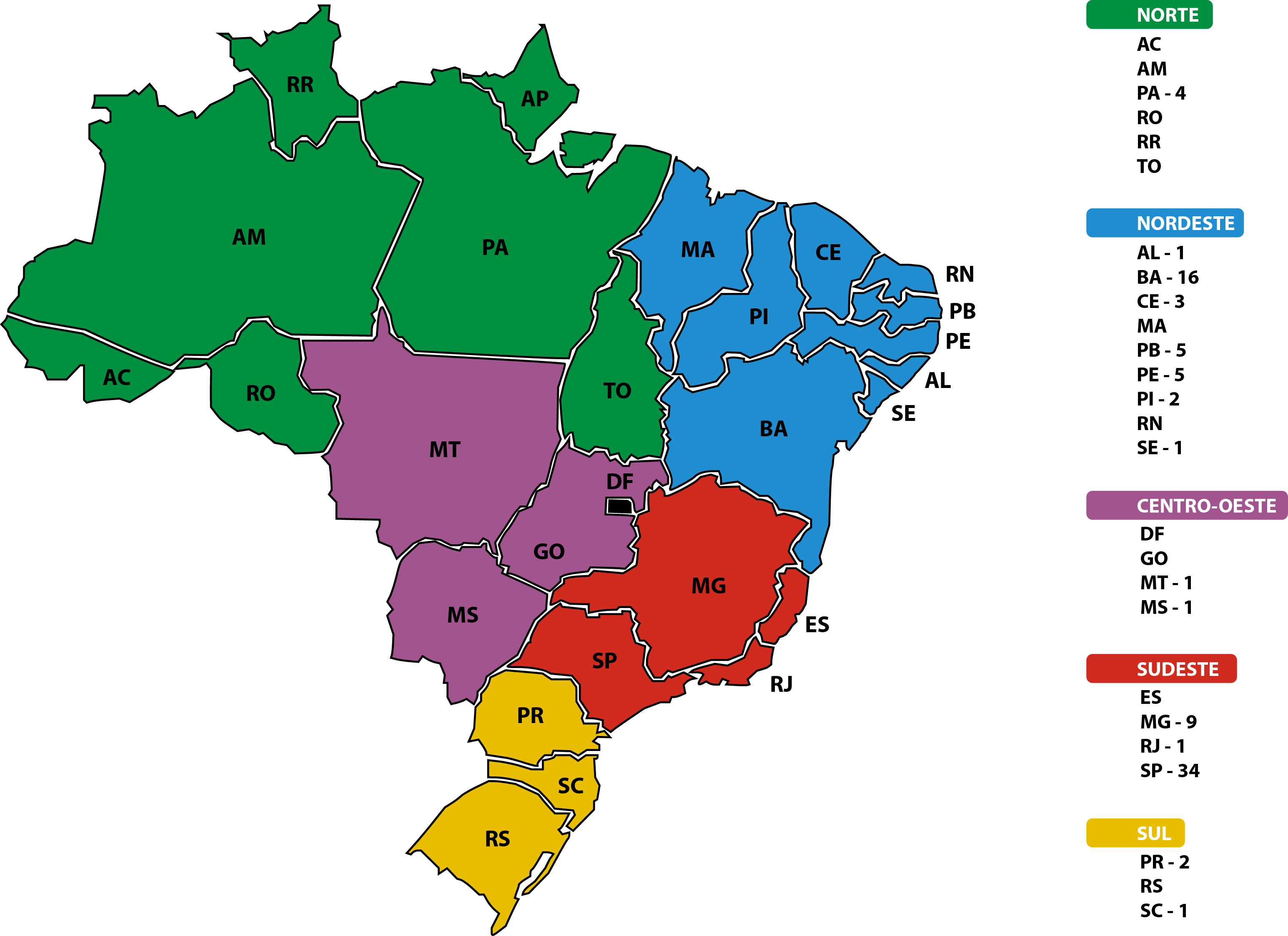
**

The Number of patients included in this study is indicated along with their respective states of origin.

PA, Pará; AL, Alagoas; BA, Bahia; CE, Ceará; PB, Paraíba; PE, Pernambuco; PI, Piauí; SE, Sergipe; MT, Mato Grosso; MS, Mato Grosso do Sul; MG, Minas Gerais; RJ, Rio de Janeiro; SP, São Paulo; PR, Paraná; SC, Santa Catarina.

**Supplementary Figure 2 – Eletrophoresis of amplicons in agarose gel.**

**
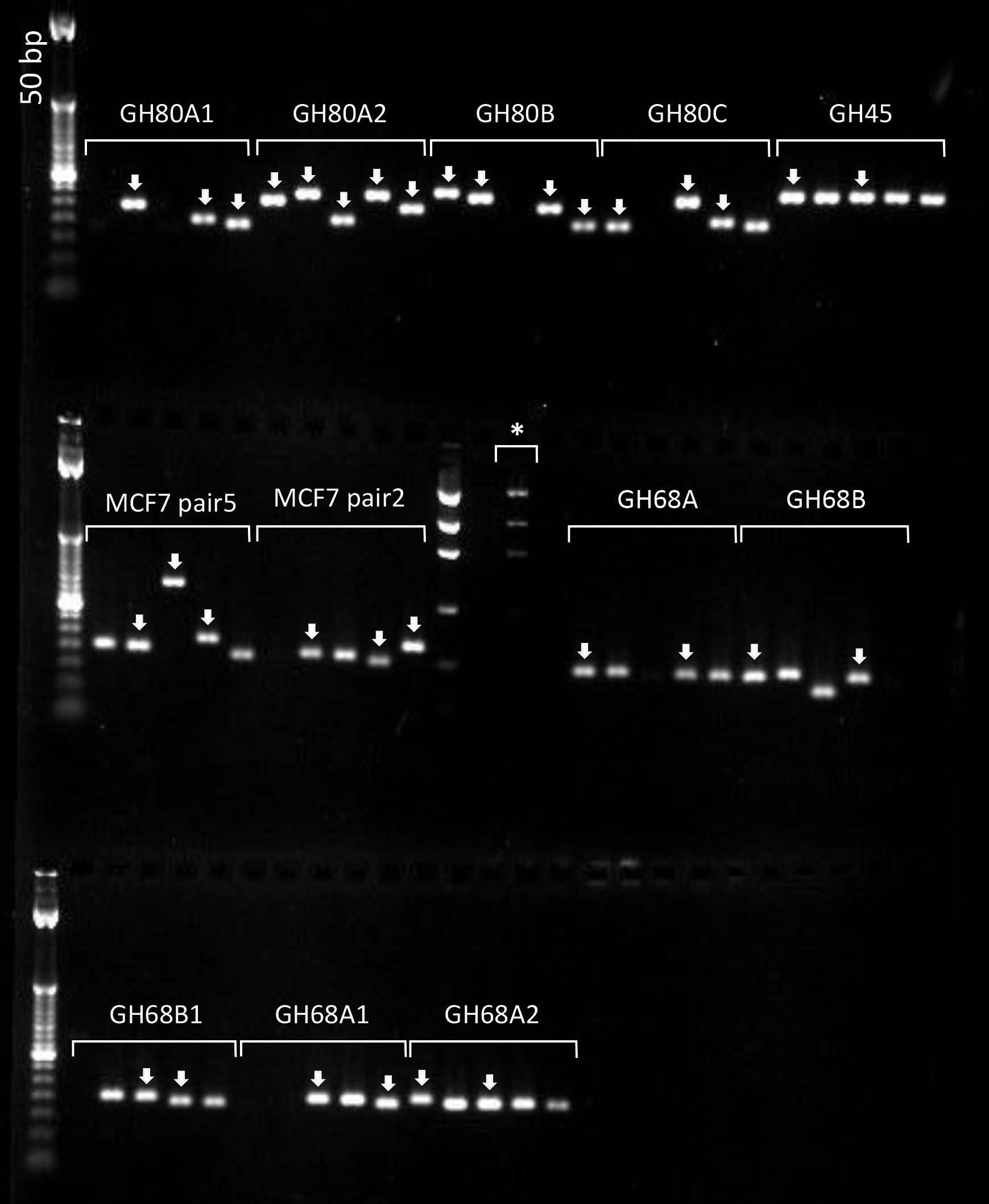
**

RNA was extracted from tumor material and after RT-PCR, amplicons detected in agarose gels were cloned, amplified in bacteria and sequenced. Arrows indicate the clones further sequenced.
